# Estimating Exposure Risk to Guide Behaviour During the SARS-COV2 Pandemic

**DOI:** 10.3389/fdgth.2021.655745

**Published:** 2021-05-28

**Authors:** Barry Smyth

**Affiliations:** Insight SFI Research Centre for Data Analytics, University College Dublin, Dublin, Ireland

**Keywords:** SARS-CoV2 (COVID- 19), estimating exposure, communicating exposure risk, pandemic data analysis, COVID-19 exposure risk case-study

## Abstract

The end of 2020 and the beginning of 2021 was a challenging time for many countries in Europe, as the combination of colder weather, holiday celebrations, and the emergence of more transmissible virus variants conspired to create a perfect storm for virus transmission across the continent. At the same time lockdowns appeared to be less effective than they were earlier in the pandemic. In this paper we argue that one contributing factor is that existing ways of communicating risk—case numbers, test positivity rates, hospitalisations etc.—are difficult for individuals to translate into a level of personal risk, thereby limiting the ability of individuals to properly calibrate their own behaviour. We propose an new more direct measure of personal risk, *exposure risk*, to estimate the likelihood that an individual will come into contact with an infected person, and we argue that it can play an important role, alongside more conventional statistics, to help translate complex epidemiological data into a simple measure to guide pandemic behaviour. We describe how exposure risk can be calculated using existing data and infection prediction models, and use it to evaluate and compare the exposure risk associated with 39 European countries.

## 1. Introduction

Although multiple, positive, vaccine trial results created a strong sense of optimism toward the end of 2020, continued calls for people to observe recommended mitigation practices (social distance, mask wearing, hand hygiene, ventilating enclosed spaces etc.) were a constant reminder that the SARS-COV2 virus remained a clear and present danger, as many countries continued to struggle to contain it (1–9). The potential for a new and even more severe wave of infections in the northern hemisphere was widely signalled, and the combination of colder weather, holiday celebrations, and the emergence of more transmissible variants of the virus (10–12) all conspired to create a perfect storm for virus transmission by the end of 2020.

Moreover, recent reports have pointed to increased mobility levels and a gradual decrease in adherence to regulations during the latest lockdowns, compared with the first wave during early 2020 (13, 14). While some have been quick to cite “lockdown fatigue” as an explanation, the evidence for this has been lacking (15–17). For example, despite claims of lockdown fatigue in the UK, recent research has concluded that there is little evidence of a decreasing trend in compliance that could be framed as a form of behavioural fatigue; see (16). However, the same study does acknowledge substantial capability, opportunity, and motivational factors that could be contributing to lower levels of adherence. Whether this turns out to be a meaningful distinction remains to be seen. Either way, a recent report by the World Health Organization (18) suggests ways for governments to address such adherence problems—which, incidentally, it frames in terms of “pandemic fatigue”—highlighting the importance of allowing people to live their lives by enabling them to reduce their levels of personal risk by using clear and simple forms of communication to guide behaviour.

One of the key challenges facing health officials and governments, in communicating the current state of the virus, has been a reliance on a parade of complicated epidemiological statistics—normalised case counts, the R number, test positivity rates, doubling rates—which can be difficult for the public to digest and even harder to translate into a level of personal risk. Consequently, our aim in this paper, is to propose a more direct measure of personal risk, which we call *exposure risk*, as an estimate of the likelihood that someone will be *exposed* to an infected individual on a per contact basis. This is effectively the probability that a single contact will turn out to be infected, while remaining silent on whether such a contact will result in actual transmission. We propose that this metric has a valuable role to play in helping the public to better interpret conventional pandemic statisitics, because it relates to their personal level of risk. As such, this metric is one of a growing number of tools and techniques that have been designed and developed during the pandemic to help governments and health officials to monitor and manage outbreaks (19–25). In what follows we will formally define the exposure risk metric and describe how it can be calculated from existing data sources. We then go on to present the results of an evaluation of exposure risk across Europe, paying particular attention to the differences between the early and later periods of the pandemic.

## 2. Materials and Methods

In this section we formally define our proposed estimate of exposure risk before describing how we evaluate it in the context of a comparative analysis of European countries using publicly available datasets.

### 2.1. Defining Exposure Risk

Exposure risk is the probability that a single contact will expose an individual to the virus. This can be estimated as a per capita measure of the number of infected individuals *at large* in the community at a given point in time; see Equation (1).


(1) $$\begin{array}{l} E=\frac{\text{number of non-isolating infectious individuals}}{\text{population size}} \end{array}$$

An exposure risk of 0.01 means that 1 in 100 (1%) of the population are infected and at large in the community. This does not mean that there is a 1% chance of becoming infected from such a contact, as there are many other factors that determine whether transmission occurs (mask wearing, contact time, distance, environment, ventilation, variant transmissibility etc.). However, it does allow an individual to form a more intuitive understanding of the likelihood that they will come into contact with an infected individual during the course of a day or a week. And since exposure risk is additive across contacts, a 1% exposure risk becomes a 10% daily risk for an individual with 10 (independent) contacts per day, all other things being equal.

Estimating the exposure risk is not straightforward. For a start, reported case numbers do not provide an accurate account of true infections; many infections are mild or even asymptomatic (26, 27) and, as such, they are less likely to present for, or be identified by, testing, especially when testing capacity is limited or close to capacity. In response, a variety of models have been developed to predict the number of true infections associated with a given country or location (28–30). These models work by using data, such as confirmed cases and deaths, testing rates, epidemiological knowledge about SARS-COV2 etc. to estimate true infections and other important measures. Here we use 4 such models: the Imperial College model, the Institute for Health Metrics and Evaluation (IHME) model, the Youyang Gu model, and the London School of Hygiene and Tropical Medicine (LSHTM). They were chosen as representatives of the type of models that have been widely used during the pandemic and because their predictions are available for a wide range of countries; further details about these models and their data is available in (31).

Each model, *m* ∈ *M*, produces an estimate for the number of infections, *m*(*d, l*), on date *d* in location *l*, and in this work we generate an ensemble prediction based on the average of the predictions of the individual models; see Equation (2)


(2) $$\begin{array}{l} i\left(d,l\right)=\frac{\underset{m\in M}{\sum }m\left(d,l\right)}{\left|M\right|} \end{array}$$

The difference between this estimate of infections and the number of confirmed cases is the number of *undetected infections*, *u*(*d, l*), in location *l* on date *d*; see Equation (3). Note the number of cases, *c*(*d, l*), is defined as the number of reported cases in *l* on date *d*. Since the reporting date of cases usually lags their infection date, it may be necessary to align infections and cases by shifting cases by an estimate of this lag. For this study we use a 10-day lag, which is unlikely to be correct in all situations, but the results have been determined to be not sensitive to minor variations in this lag; see section 4.


(3) $$\begin{array}{l} u\left(d,l\right)=i\left(d,l\right)-c\left(d,l\right) \end{array}$$

Next, we need to calculate the number of undetected infections that are active on a particular date, given that infected individuals remain infected for a number of days. Thus, we need to calculate the *prevalence* of undetected infections. For SARS-COV2, prevalence is usually calculated as the 14-day total of cases; this is also the recommended isolation time for (suspected) infected individuals. Thus, the prevalence of undetected infections, *U*(*d, l*), for location *l* on date *d*, is given by Equation 4.


(4) $$\begin{array}{l} U\left(d,l\right)=\underset{d-13\;\leq d_{i}\;\leq d}{\sum }u\left(d_{i},l\right) \end{array}$$

In order to estimate the exposure risk we make two further assumptions: (1) that confirmed cases *do not* present an exposure risk, because they will be isolating; and (2) that undetected infections *do* present an exposure risk, because they may be circulating in the community, unaware they are infected. This is obviously a simplification of the reality (see section 4): cases will be contagious for a time before they are confirmed; not everyone will isolate as or when they should; many undetected cases may be asymptomatic or mild, thereby presenting a lesser risk; and some undetected cases may self-isolate if they are feeling unwell. Then, exposure risk is defined as the number of undetected infections as a fraction of population; see Equation (5).


(5) $$\begin{array}{l} E\left(d,l\right)=\frac{U\left(d,l\right)}{pop\left(l\right)} \end{array}$$

It is useful to consider a *relative* version of exposure risk too, by calculating the current exposure risk as a fraction of the *peak* exposure risk at some earlier point in time, such as during the first wave of infections when many countries locked-down hard; see Equations (6–8). For example, the exposure risk in the Netherlands at the end of 2020 (0.005) was more than 3-times lower than its peak exposure risk in early 2020, whereas Austria's late-2020 exposure risk (0.027) was more than 6-times higher than it was in early 2020. This relative exposure risk may help people to calibrate their actions relative to their springtime behaviour, which defined the pandemic-level behaviour of many.


(6) $$\begin{array}{l} PeakDate\left(start,end,l\right)=\underset{d}{\mathrm{argmax}}E\left(d,l\right)\;\forall d\;:start\leq d\leq end \end{array}$$

(7) P(d,start,end,l)=E(PeakDate(start,end,l),l)

(8) $$\begin{array}{l} relE\left(d,start,end,l\right)=\frac{E\left(d,l\right)}{P\left(d,start,end,l\right)} \end{array}$$

### 2.2. Datasets and Approach

We evaluate exposure risk for 39 countries in Europe throughout the pandemic so far using public data covering the period from March 1, 2020 to February 28, 2021; confirmed cases and population data are available in (32) and infection prediction data are available in (31). We limit our analysis to European countries primarily because of their geographical proximity, which is a significant factor in the temporal pattern of the infection waves that have occurred so far. For each country and date we calculate its mean daily exposure risk and also its peak exposure risk during the *early period* (March 1–September 26 2020 inclusive) and the *late period* (October 2 2020–February 28 2021, inclusive); each period is 150 days in duration.

## 3. Results

We present the results of this analysis in two parts. First, we begin with a case-study of the daily exposure risk for Ireland and the Netherlands during the pandemic before presenting the results obtained for the full set of 39 European countries.

### 3.1. Exposure Risk Case-Study

Ireland is an interesting case-study, because it went from being one of the best performing countries in Europe, in terms of daily cases per capita, to one of the worst in the world, all in a matter of only a few short weeks. The country experienced an explosion of cases over the Christmas period after having one of the lowest case-counts in Europe just weeks before; if there was ever a need to communicate exposure risk more effectively to people, then Ireland needed it.

**Figure 1:**
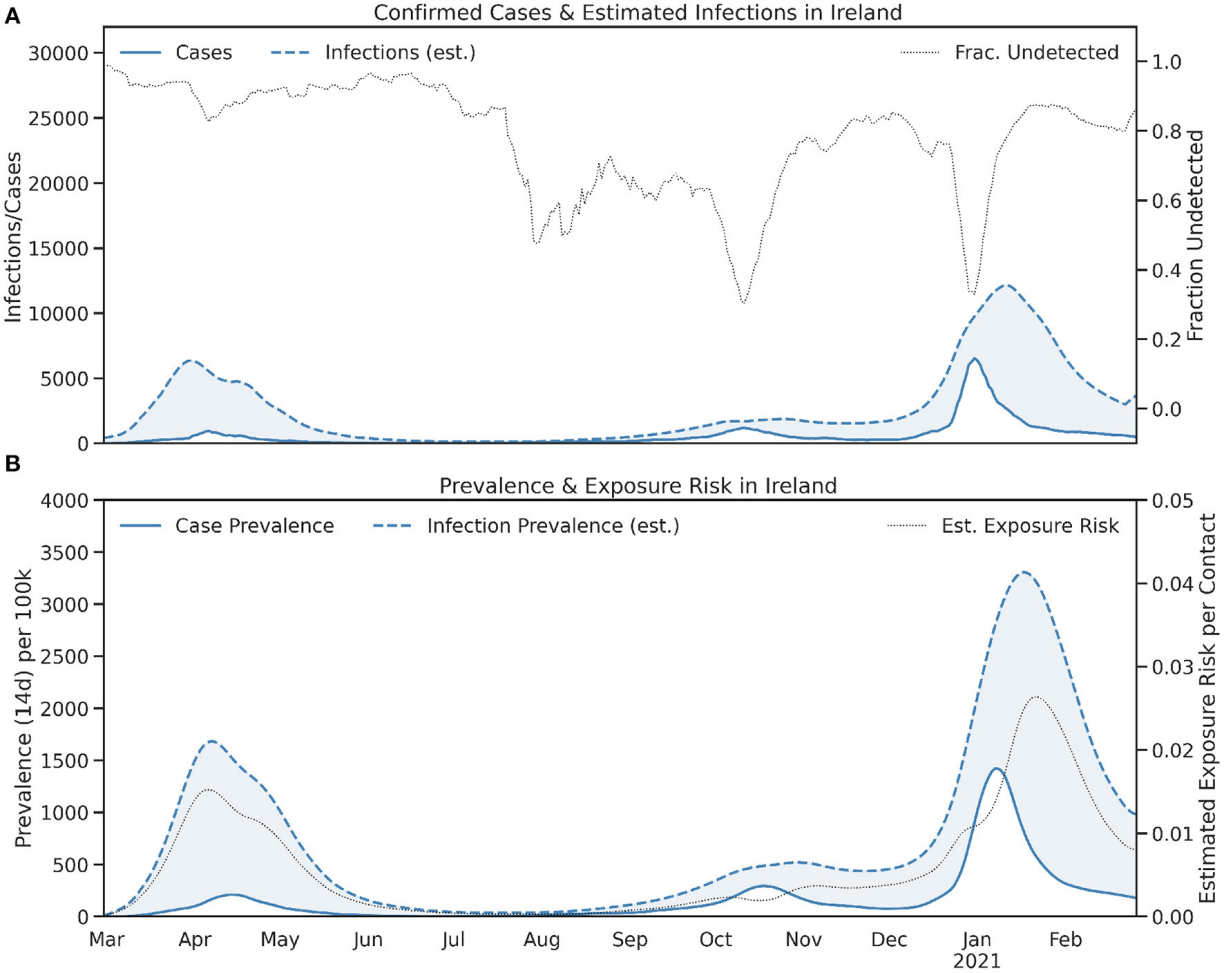
The **(A)** daily and **(B)** 14-day totals (prevalence) of confirmed cases and estimated infections in Ireland. In **(A)** the fraction of undetected infections and in **(B)** the estimated exposure risk, are also shown.

Figure 1A shows the total number of daily cases and the estimated infections. The shaded region is the difference between these cases and infections—that is, the number of undetected infections—and it is striking to see just how large this difference has been at different points in the pandemic. For example, at the peak of the first wave in April, Ireland reported more than 900 cases per day, but there was an estimated peak of approximately 7,500 infections. Ireland's explosive recent wave saw cases soar to more than 5,000 cases per day, but with infections predicted to be almost 3-times this number. The dotted line in Figure 1A shows the number of undetected infections as a fraction of total infections and it is frequently above 0.8 for Ireland. This is consistent with similar data elsewhere (33) and helps to highlight the scale of the difference between confirmed cases and true infections, even in countries with mature testing infrastructures.

The estimated infections indicate that Ireland's peak cases in October, while higher than the corresponding peak in April, was associated with fewer infections than in April. This suggests Ireland's improved testing infrastructure helped it to identify a much greater fraction of infections (approximately 50–60%) in October compared to the 10–20% of infections that were identified in April. Thus, while Ireland's October case numbers led it into a second, strict lockdown in November it is noteworthy that true infections in October reached lower levels than in April. The same cannot be said for its cases or infections during late December and January, however.

Figure 1B shows the corresponding 14-day prevalence, for cases and infections; the prevalence of undetected infections is represented by the shaded region. The corresponding estimated exposure risk is also shown. At the end of 2020 even though Ireland's recent infection prevalence was much higher than it was in April 2020, the corresponding exposure risk was only marginally higher than the peak exposure risk from April, in part due to an improved ability to find cases in late 2020. Unfortunately, even Ireland's enhanced testing infrastructure was unable to cope with the growth of cases at the end of 2020 and the exposure risk peaked at 0.026 (2.6%) in late January 2021 compared to 0.019 in April 2020; thus the relative exposure risk is Ireland was approximately 37% higher in January 2021 compared with the April 2020 wave.

**Figure 2:**
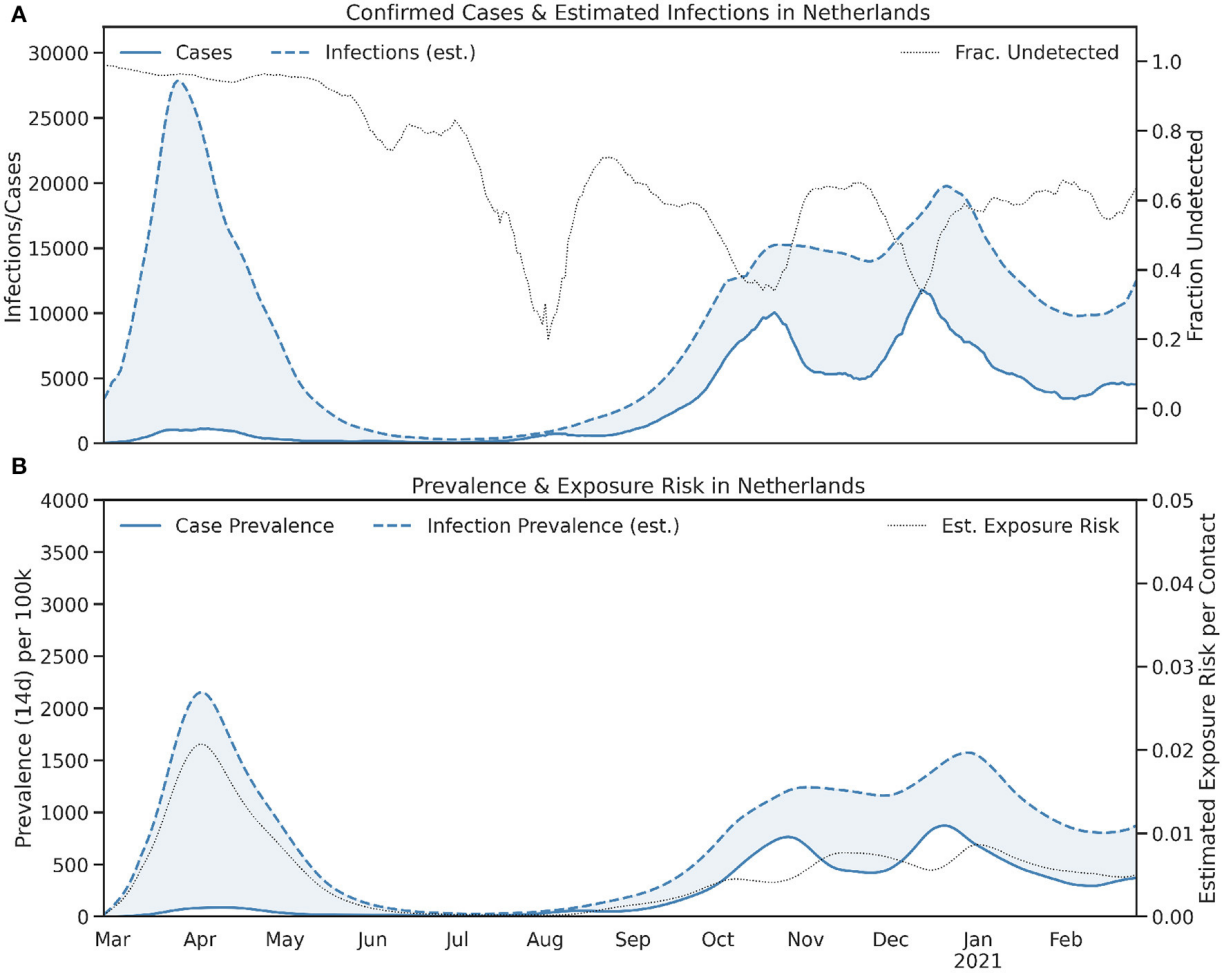
The **(A)** daily and **(B)** 14-day totals (prevalence) of confirmed cases and estimated infections in the Netherlands. In **(A)** the fraction of undetected infections and in **(B)** the estimated exposure risk, are also shown.

Figure 2 shows the equivalent graphs for the Netherlands. As was the case in Ireland, the Netherlands suffered from a significant outbreak in April 2020, with a large sustained outbreak in late 2020 and early 2021. However, the testing infrastructure in the Netherlands was able to cope with this wave and, unlike Ireland, exposure risk remained low (<1%) throughout.

### 3.2. Comparing Cases, Infections, and Exposure Risk in Europe

**Figure 3:**
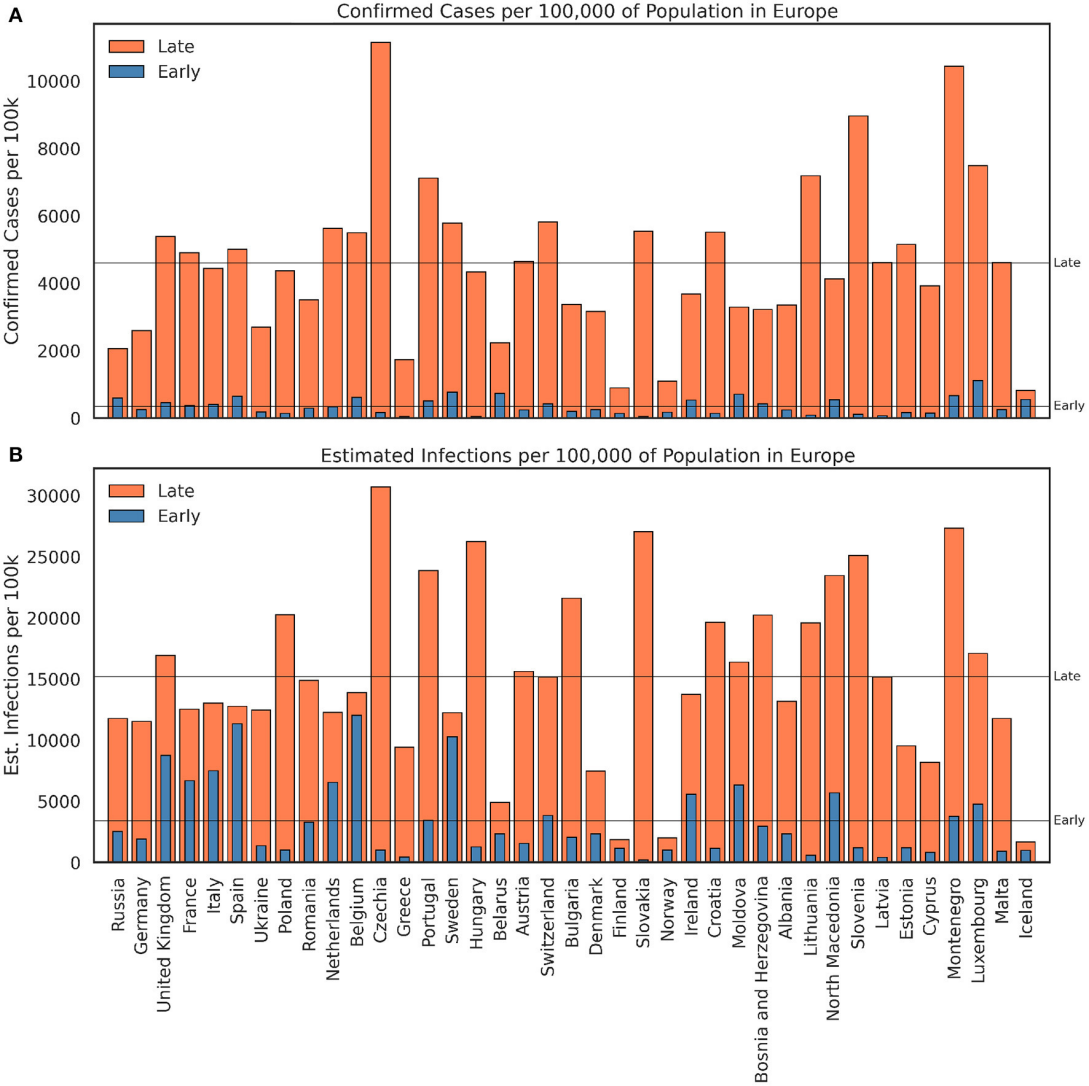
The total number of **(A)** confirmed cases and **(B)** estimated infections, per 100,000 of population, during the *early period* (March–September 2020) and the *late period* (October 2020 - February 2021) for 39 European countries.

Broadly speaking many European countries have seen a similar pattern of cases and infections over the course of 2020. The bar charts in Figure 3 show (A) the *total* number of confirmed cases, per 100,000 of population, and (B) the estimated infections, per 100,000 of population, for each country in Europe during the *early* and *late* periods; countries are ordered, left to right, in descending order of population size.

It is clear in Figure 3A that the confirmed case counts associated with the late period are now significantly higher than the early period for every country. On average, European countries confirmed just under 350 cases per 100 k in the early period, compared with just under 4,600 cases per 100 k in the late period, a relative increase of more than 13x. In part this can be explained by significant improvements in testing capability—more testing means more confirmed cases—although this is far from a complete explanation since the estimated infections tell a similar story in Figure 3B, indicating that infection rates have also been higher in the late period. In fact, there are no countries with fewer infections during the late period compared with the early period; on average the late period generated 15,193 infections per 100 k compared with 3,394 infections per 100k in the early period, greater than a 4x increase. These differences in mean cases and infections, between the early and late periods, are statistically significant, based on a one-sided *t* test; *t*_(76)_ = – 11.36, *p* < 0.001 for cases and *t*_(76)_ = – 9.44, *p* < 0.001 for infections. Thus, we can state with some confidence that the late period has been more severe in Europe, because it has resulted in significantly more cases and more infections, even allowing for improvements in testing.

**Figure 4:**
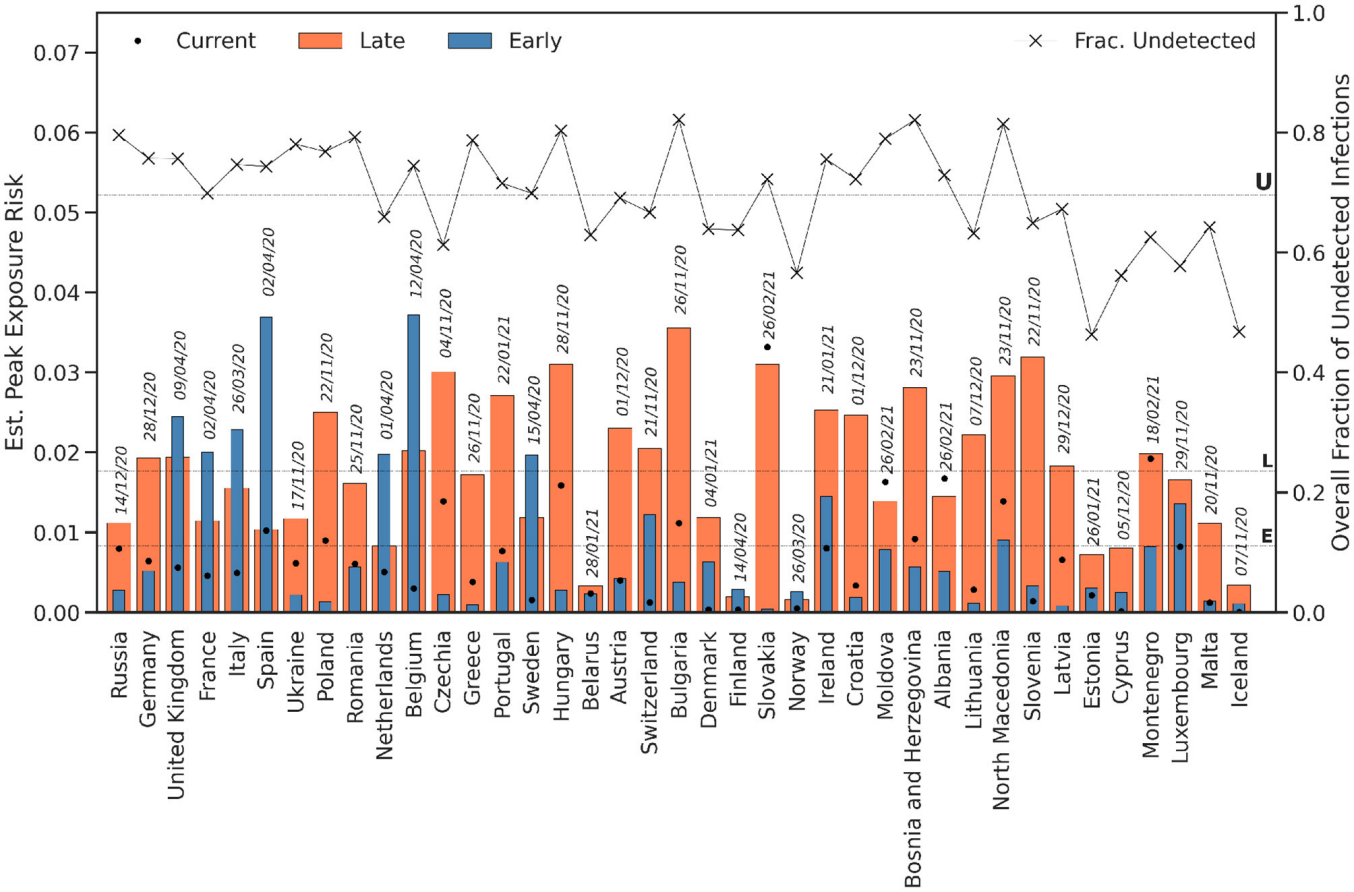
The peak exposure risk (bars) for the early and late periods, and the current exposure risk, for countries in Europe. The line graph presents the average fraction of undetected infections for each country during the pandemic so far. The horizontal lines correspond to the mean (peak) exposure risks—for the early (*E*) and late (*L*) periods—and the mean fraction of undetected infections (*U*). The dates annotating the bars indicate the date of the peak exposure risk for that country for the pandemic so far.

Notably, this does not necessarily mean that the exposure risk is correspondingly greater in the late period, because exposure risk depends on the fraction of *undetected* infections, rather than the actual number of cases or infections. Since testing infrastructure has improved, we should expect fewer undetected infections, all other things being equal, and thus a relative improvement in the exposure risk. We can see this in Figure 4, which compares the peak exposure risk for countries between the early and late periods. There are nine countries (the UK, France, Italy, Spain, the Netherlands, Belgium, Sweden, Finland, and Norway) whose peak exposure risk was higher in the early period than in the late period. None had more infections in the early period, but they did have a greater proportion of undetected infections, and hence a higher exposure risk. On average, the peak exposure risk in the early period was 0.008 (0.8%), compared with 0.018 (1.8%) in the late period. That's more than twice the peak exposure risk in the late period but from 4x the infections and 13x the confirmed cases.

Figure 4 also indicates the (current) exposure risk for each country at the end of the late period (February 28, 2021), using a single filled dot marker, and only 4 countries (Slovakia, Moldova, Albania, and Montenegro) were still peaking at that time. For example, Slovakia, which managed to contain infections very well in the early period (with very low peak exposure risk levels of < < .01) was suffering from a peak exposure risk >3% at the end of the late period. For completeness, Figure 4 also shows the fraction of undetected infections for each country in the pandemic so far, which varies from <0.5 (Estonia and Iceland) to almost 0.9 (Bulgaria, Boznia & Herzegovnia, and North Macedonia); on the average fraction of undetected infections overall was 0.70 (U).

## 4. Discussion

We have described an approach to estimating the risk of SARS-COV2 exposure (per contact) based on the prevalence of undetected infections per capita. It is important to underscore that this measure is developed to help communicate the level of risk to the general public, rather than as a forecasting tool. Consequently, the level of accuracy of this metric is less important that its explanatory value; although obviously it is important for it to present a reasonably accurate estimate of personal risk if it is to be accepted and trusted. In this section we discuss some of the factors that are important in understanding the likely accuracy of this exposure risk estimate, and some of the ways that the estimate might be improved in the future.

To calculate exposure risk we made a number of assumptions that are worth revisiting. One important assumption was the availability of sufficiently accurate estimates of true infections. We based our estimates on the predictions of four prominent infection models, which have been used and relied upon throughout this pandemic and some recent studies have concluded that such models exhibit reasonable levels of prediction accuracy (34, 35). Moreover, our ensemble approach, based on the average of these underlying models, can be expected to produce more robust estimates than if we relied upon any single model. While a detailed analysis of the accuracy of these models is beyond the scope of this paper, it is worth noting that the resulting fraction of undetected infections produced is in broad agreement with the seroprevalence data that does exist; see for example, (36).

Another assumption made was that our estimate of exposure risk should be based on the per capita, 14-day prevalence of undetected infections. The rationale for this is that confirmed cases will be isolating and therefore should not present a significant exposure risk, while undetected cases can be expected to be circulating in the community. There are a number of points worth noting regarding the validity of this assumption and the accuracy of the resulting estimate:

1. Most confirmed cases will have been contagious before they developed symptoms and, therefore, before they were confirmed (37), and many of these will have been circulating in the community. By excluding confirmed cases we are underestimating the exposure risk.
2. Recent studies suggest that about a fifth of infections are genuinely asymptomatic (37, 38) and there is some evidence to support the idea that asymptomatic cases are likely to be less contagious than symptomatic ones (38). It is reasonable to assume that asymptomatic cases are more likely to be undetected and thus by including these undetected cases we may be overestimating the exposure risk. On the other hand it is also worth remembering that exposure risk deals with exposure rather than transmission so one could dismiss the lower contagiousness of asymptomatic cases as moot.
3. A majority of undetected cases must be symptomatic, however—if up to 80% of infections are undetected but only 20% are asymptomatic, then on average about 60% of infections are symptomatic and undetected—but presumably they are likely to be milder on average than the typical confirmed case and, again, there is some evidence that milder cases can be less contagious than more severe cases (39), because they shed the virus for a shorter period. Once again this may contribute to an over-estimation of transmission risk.

Undoubtedly, there is potential for error in our approach to estimating exposure risk, but the competing nature of these sources of error may limit its extent in practice. Indeed it may be possible to fine-tune the exposure estimate further too, for example by developing a weighted model which combines confirmed and undetected infections, using differently weighted exposure periods. Either way, the present model provides a useful and straightforward starting point that is likely to be sufficiently accurate and robust as a practical public-facing measure. After all, the primary objective is to help people to understand the level of risk (e.g., low, moderate, and high) so that they can calibrate their behaviour, and, as such, a high degree of precision is less important.

We assumed a fixed 10-day lag between case reporting and infection dates and this is another potential source of error. This could be addressed by more accurately accounting for this lag, which is likely to change from location to location depending on factors such as testing capacity. Indeed, it is likely that much more precise estimates of the infection dates of cases are readily known as a result of testing and therefore could be incorporated on a location-by-location basis. Related to this is the issue that not all countries report their cases in a consistent fashion, and the public case data occasionally includes adjustments to rectify such issues. In general these issues appear to be rare and mostly associated with the early months of the pandemic, although it is likely too that case reporting will have been less accurate during the peaks of the late periods when the testing infrastructure of many countries was under great stress.

The approach so far assumes exposure risk to be evenly distributed across a population. This is very unlikely to be the case and studies have highlighted significant variation in seroprevalence at a regional level (40, 41) and among different ethnic or socio-demographic groups (42, 43). However, this is not a limitation of the approach proposed per se, as much as it is an artefact of our choosing to work with country-level data. Certainly, by working with more fine-grained data (tests, cases, and deaths) it will be possible to generate better estimates of infections and exposure risks for different local regions or among different socio-demographic clusters or age groups. Such data will be available within the data repositories of most health systems and, as such, should be straightforward to use to generate these more precise estimates of exposure risk.

It is worth revisiting why we should go to the trouble of calculating the exposure risk metric in the first place. The contrary position might be that, while it is a meaningful metric, from the point of view of helping an individual to calibrate their level of personal risk, it would be easier to use cases or test positivity rates in much the same way. The point is that neither confirmed cases nor test positivity rates correlate very closely with the exposure risk estimate. For example, the *r*^2^ between confirmed cases (per 100k) and exposure risk during the pandemic, averaged across the 39 countries, is just 0.44 and the corresponding *r*^2^ value for test positivity rates is 0.55. In other words, neither of these metrics on their own is sufficient to accurately estimate exposure risk. Not surprisingly, the estimate of the number of true infections is much more closely correlated with exposure risk (*r*^2^ = 0.88) but even then it is not perfect, and after all, it is a simple calculation to transform infections into exposure risk to produce a far more relatable measure for the general public.

Finally, it is important to recognise that this estimate of exposure risk is not designed to predict the risk of transmission per se, which will depend on other factors and behaviours, such as mask wearing or the prevalence and transmissibility of new virus variants. It may be possible to estimate the likelihood of transmission, given exposure, based on an analysis of the effectiveness of masks, social distancing, and variant transmissibility, but this is beyond the score of this work.

## 5. Conclusions

As many countries, in Europe and elsewhere, continue to impose restrictions to control the recent wave of infections, it is becoming increasingly difficult to contain this virus, especially in the face of emerging variants that are more transmissible. The World Health Organisation has highlighted the importance of enabling people to live their lives safely by empowering them to reduce their levels of personal risk using clear communication messages based upon simple and intuitive metrics to guide their behaviour. We propose exposure risk as one such metric. We describe how it can be readily calculated from existing sources of public pandemic data, and compare the exposure risk of 39 European countries during the pandemic so far. By directly estimating how likely an individual is to be exposed to the virus, it can be argued that exposure risk provides a much more meaningful measure with which to guide behaviour.

## Data Availability Statement

All data used in this study are publicly available and cited in the main text. Further questions can be directed to the author.

## Author Contributions

BS is the originator of the ideas presented in this manuscript and has carried out of all of the work associated with it.

## Conflict of Interest

The author declares that the research was conducted in the absence of any commercial or financial relationships that could be construed as a potential conflict of interest.

## References

[1] Anderso R M Hollingsworth T D Baggaley R F Maddren R Vegvari C. Covid-19 spread in the uk: the end of the beginning? Lancet (2020) 396:587–90. 10.1016/S0140-6736(20)31689-5 32758428 PMC7398685

[2] Dahlberg M Edin P-A Grönqvist E Lyhagen J Östh J Siretskiy A. Effects of the covid-19 pandemic on population mobility under mild policies: Causal evidence from sweden. arXiv Preprint arXiv:2004.09087. (2020).

[3] Engle S Stromme J Zhou A. Staying at home: mobility effects of covid-19. Available at SSRN (2020).

[4] Hale T Petherick A Phillips T Webster S. Variation in government responses to covid-19. Blavatnik School of Government Working Paper (2020). 31.

[5] Verity R Okell L C Dorigatti I Winskill P Whittaker C Imai N. Estimates of the severity of coronavirus disease 2019: a model-based analysis. Lancet (2020) 20:669–77. 10.1016/S1473-3099(20)30243-7 32240634 PMC7158570

[6] Viner R M Russell S J Croker H Packer J Ward J Stansfield C. School closure and management practices during coronavirus outbreaks including covid-19: a rapid systematic review. Lancet Child Adolescent Health (2020) 4:397–404. 10.1016/S2352-4642(20)30095-X 32272089 PMC7270629

[7] Kumar M Gupta S Kumar K Sachdeva M. Spreading of covid-19 in indiaindia, italy, japan, spain, uk, uss: a prediction using arima and lstm model. Digit Govern (2020) 1:1–9. 10.1145/3411760

[8] Zhang J Litvinova M Liang Y Wang Y Wang W Zhao S. Changes in contact patterns shape the dynamics of the COVID-19 outbreak in China. Science(2020) 368:1481–6. 10.1126/science.abb8001 32350060 PMC7199529

[9] Zhang S Diao M Y Duan L Lin Z Chen D. The novel coronavirus (SARS-CoV-2) infections in China: prevention, control and challenges. Intensive Care Med (2020) 46:591–3. 10.1007/s00134-020-05977-9 32123989 PMC7079863

[10] Hou Y J Chiba S Halfmann P Ehre C Kuroda M Dinnon K H Leist S R. Sars-cov-2 d614g variant exhibits efficient replication *ex vivo* and transmission *in vivo*. Science (2020) 370:1464–8. 10.1101/2020.09.28.317685 33184236 PMC7775736

[11] Volz E Hill V McCrone J T Price A Jorgensen D O' Toole Á. Evaluating the effects of sars-cov-2 spike mutation d614g on transmissibility and pathogenicity. Cell (2020) 184:64–75.e11. 10.1016/j.cell.2020.11.020 33275900 PMC7674007

[12] Tegally H Wilkinson E Giovanetti M Iranzadeh A Fonseca V Giandhari J. Emergence and rapid spread of a new severe acute respiratory syndrome-related coronavirus 2 (sars-cov-2) lineage with multiple spike mutations in south africa. medRxiv (2020).

[13] Folmer C R Brownlee M Fine A Kuiper M E Olthuis E Kooistra E B. Social distancing in america: Understanding long-term adherence to covid-19 mitigation recommendations. PsyArXiv (2020). 10.1371/journal.pone.0257945 PMC8462713 34559863

[14] Masten A S Motti-Stefanidi F. Multisystem resilience for children and youth in disaster: Reflections in the context of covid-19. Advers Resil Sci (2020) 1:95–106. 10.1007/s42844-020-00010-w 32838305 PMC7314620

[15] Harvey N. Behavioral fatigue: real phenomenon, naïve construct, or policy contrivance? Front Psychol (2020) 11:589892. 10.3389/fpsyg.2020.589892 33224078 PMC7674166

[16] Michie S West R Harvey N. The concept of “fatigue” in tackling covid-19. bmj (2020) 371:m4171. 10.1136/bmj.m4171 33139254

[17] Mahase E. Covid-19: was the decision to delay the uk's lockdown over fears of “behavioural fatigue” based on evidence? BMJ. (2020) 370:m3166. 10.1136/bmj.m3166 32769080

[18] Organization W H. Pandemic fatigue: reinvigorating the public to prevent covid-19: policy framework for supporting pandemic prevention and management: revised version november 2020. (2020) Technical report, World Health Organization. Regional Office for Europe.

[19] Dong E Du H Gardner L. An interactive web-based dashboard to track COVID-19 in real time. Lancet Infect Dis (2020) 20:533–4. 10.1016/S1473-3099(20)30120-1 32087114 PMC7159018

[20] Gupta R Pandey G Chaudhary P Pal S K. Machine learning models for government to predict covid-19 outbreak. Digit Govern (2020) 1:1–6. 10.1038/s41746-020-00372-6

[21] Röddiger T Beigl M Dörner D Budde M. Responsible, automated data gathering for timely citizen dashboard provision during a global pandemic (covid-19). Digit Gov Res Pract (2020) 2. 10.1145/3428471

[22] Sheikh A Sheikh A Sheikh Z Dhami S Sridhar D. What's the way out? potential exit strategies from the covid-19 lockdown. J Glob Health (2020) 10:010370. 10.7189/jogh.10.010370 32566161 PMC7296206

[23] Smyth B. Lockdowns & rebounds: a data analysis of what happens next. Digit Govern Res Pract (2020) 1:1–7. 10.1145/3411762

[24] Smyth B. Estimating the fatality burden of sars-cov2. Digit Govern Res Pract. (2020) 2:1–8. 10.1145/3436997

[25] Yuan J Li M Lv G Lu Z K. Monitoring transmissibility and mortality of covid-19 in europe. Int J Infect Dis (2020) 20:1878:3511. 10.1016/j.ijid.2020.03.050 32234343 PMC7102547

[26] Nikolai L A Meyer C G Kremsner P G Velavan T P. Asymptomatic sars coronavirus 2 infection: invisible yet invincible. Int J Infect Dis (2020) 100:112–6. 10.1016/j.ijid.2020.08.076 32891737 PMC7470698

[27] Duan S Zhou M Zhang W Shen J Qi R Qin X. Seroprevalence and asymptomatic carrier status of sars-cov-2 in wuhan city and other places of china. PLoS Negl Trop Dis (2021) 15:e0008975. 10.1371/journal.pntd.0008975 33411805 PMC7790301

[28] Böhning D Rocchetti I Maruotti A Holling H. Estimating the undetected infections in the covid-19 outbreak by harnessing capture-recapture methods. Int J Infect Dis. (2020) 97:197–201. 10.1016/j.ijid.2020.06.00 32534143 PMC7286831

[29] Menkir T F Taylor Chin J H Surface E D De Salazar P M Buckee C O Watts A. Estimating the number of undetected covid-19 cases exported internationally from all of china. medRxiv. (2020).32511613

[30] Rocchetti I Böhning D Holling H Maruotti A. Estimating the size of undetected cases of the covid-19 outbreak in europe: an upper bound estimator. Epidemiol Methods. (2020) 9. 10.1515/em-2020-0024

[31] Giattino C. How Epidemiological Models of Covid-19 Help us Estimate the True Number of Infections. (2020) Available online at: https://ourworldindata.org/covid-models.

[32] Roser M Ritchie H Ortiz-Ospina E Hasell J. Coronavirus Pandemic (Covid-19). (2020) Available online at: https://ourworldindata.org/excess-mortality-covid.

[33] Shaman J. An estimation of undetected covid cases in france. Nature. (2020) 590:38–9. 10.1038/d41586-020-03513-9 33349713

[34] Friedman J Liu P Gakidou E COVID I Team M C. Predictive performance of international covid-19 mortality forecasting models. medRxiv. (2020). 33972512 10.1038/s41467-021-22457-w PMC8110547

[35] Reich N G Cornell M Ray E L House K Le K. The zoltar forecast archive: a tool to facilitate standardization and storage of interdisciplinary prediction research. arXiv preprint arXiv:2006.03922 (2020).

[36] Russell T W Golding N Hellewell J Abbott S Pearson C A Jarvis C I. Reconstructing the global dynamics of unreported covid-19 cases and infections. BMC Med. (2020) 18:332. 10.1186/s12916-020-01790-9 33087179 PMC7577796

[37] Buitrago-Garcia D Egli-Gany D Counotte M J Hossmann S Imeri H Ipekci A M Salanti G. Occurrence and transmission potential of asymptomatic and presymptomatic sars-cov-2 infections: a living systematic review and meta-analysis. PLoS Med (2020) 17:e1003346. 10.1371/journal.pmed.1003346 32960881 PMC7508369

[38] Byambasuren O Cardona M Bell K Clark J McLaws M-L Glasziou P. Estimating the extent of true asymptomatic covid-19 and its potential for community transmission: systematic review and meta-analysis. Available at SSRN 3586675. (2020). 10.3138/jammi-2020-0030 PMC9602871 36340059

[39] Perera R A Tso E Tsang O T Tsang D N Fung K Leung Y W. Sars-cov-2 virus culture and subgenomic rna for respiratory specimens from patients with mild coronavirus disease. Emerg Infect Dis (2020) 26:2701. 10.3201/eid2611.203219 32749957 PMC7588524

[40] Bajema K L Wiegand R E Cuffe K Patel S V Iachan R. Estimated sars-cov-2 seroprevalence in the us as of september 2020. JAMA Inter Medi (2020) 181:450–60. 10.1001/jamainternmed.2020.7976 33231628 PMC7686880

[41] Demonbreun A R McDade T W Pesce L Vaught L A Reiser N L Bogdanovic E. Patterns and persistence of sars-cov-2 igg antibodies in a us metropolitan site. medRxiv. (2020). 33755598 10.1172/jci.insight.146148 PMC8262291

[42] Brüssow H. Covid-19 by numbers-infections, cases and deaths. Environ Microbiol (2020) 23:1322–33. 10.1111/1462-2920.15377 33368993

[43] Hallal P C Hartwig F P Horta B L Silveira M F Struchiner C J Vidaletti L P. Sars-cov-2 antibody prevalence in brazil: results from two successive nationwide serological household surveys. Lancet Global Health (2020) 8:e1390–8. 10.1016/S2214-109X(20)30387-9 32979314 PMC7511212

